# Hemorrhage in the Wall of Pyogenic Brain Abscess on Susceptibility Weighted MR Sequence: A Report of 3 Cases

**DOI:** 10.1155/2014/907584

**Published:** 2014-09-16

**Authors:** Krishnamoorthy Thamburaj, Amit K. Agarwal, Shyamsunder B. Sabat, Dan T. Nguyen

**Affiliations:** Department of Radiology, Penn State Milton S. Hershey Medical Center, Penn State College of Medicine, Hershey, PA 17033, USA

## Abstract

*Background and Purpose*. In pyogenic brain abscess, hemorrhage in the walls is considered exceptional. Recently, hemorrhagic changes in the walls of pyogenic abscess have been demonstrated on susceptibility weighted imaging with 3T MRI. Here, we report hemorrhagic changes in the walls of pyogenic brain abscess on susceptibility weighted imaging with 1.5T MRI. *Method*. MRI of brain was done using 1.5T MRI with diffusion weighted sequence, susceptibility weighted sequence, and other standard sequences in 3 consecutive cases of pyogenic brain abscess. Stereotactic biopsy and cultures were obtained in 2 cases. One case was treated empirically with antibiotics. *Results*. Susceptibility sequence demonstrated hemorrhage in the wall of brain abscess in all three cases. All three cases also demonstrated restricted diffusion on diffusion weighted imaging. *Conclusion*. Susceptibility weighted imaging can demonstrate hemorrhagic changes in the walls of pyogenic brain abscess on 1.5T MRI. Presence of hemorrhage in the walls of ring enhancing lesions should not automatically lead to a diagnosis of tumor.

## 1. Introduction

Hemorrhage in the wall of pyogenic brain abscess is exceptional and its presence is considered to support a diagnosis of hemorrhagic tumor [1–5]. Recently, with the advent of susceptibility weighted imaging (SWI), occurrence of hemorrhagic changes in the wall of pyogenic abscess has been reported on 3T MRI but not on 1.5T MRI [6, 7]. In this short series, we report three cases of pyogenic brain abscess demonstrating signals consistent with hemorrhage in SWI at 1.5T MRI.

## 2. Case Presentation

### 2.1. Case 1

**Figure 1:**
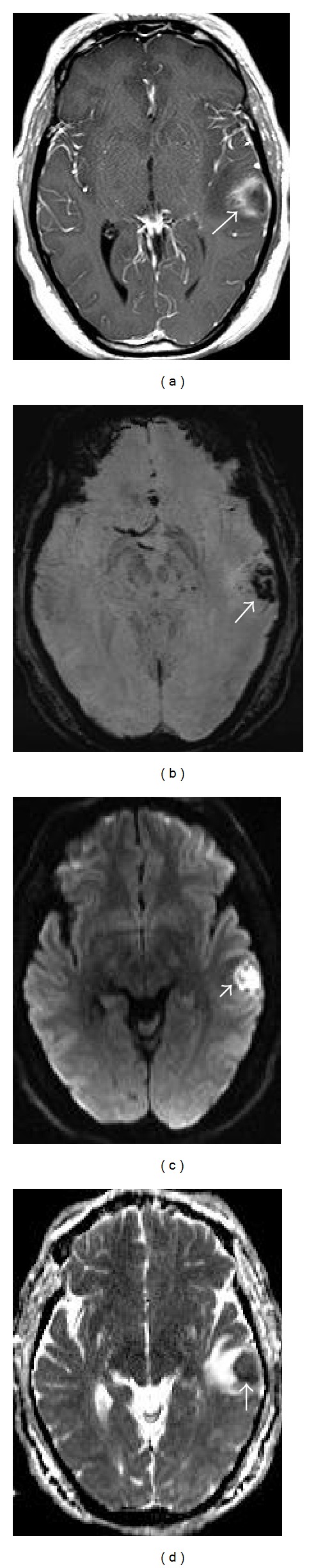
(a) Postgadolinium axial T1 SE image shows the ring enhancing wall of the left temporal abscess (arrow). Note the lack of smooth ring enhancement. (b) Susceptibility weighted image shows prominent hypointense signals (arrow) in the lesion. (c) Diffusion image demonstrates bright signals due to restricted diffusion from the pus in the center of the abscess (arrow). (d) Apparent diffusion coefficient (ADC) map shows corresponding dark signals confirming true restricted diffusion (arrow).

A 34-year-old male presented to the emergency with severe headache of 2-day duration, confusion, vomiting, and an episode of tonic clonic seizure. On neurological examination, no focal deficits were identified. Laboratory investigation revealed a total count of 8.47 K/*μ*L and a platelet count of 229 K/*μ*L. Coagulation profile was normal. An emergent noncontrast CT head revealed a hypodense lesion in the left temporal lobe with no evidence of hemorrhage or calcification. MRI brain was obtained with 1.5T MRI 14 hours later. Along with routine pre- and postcontrast T1 SE, T2 FSE, FLAIR, and diffusion imaging, susceptibility weighted sequence was obtained with parameters TR49 mS, TE40mS, ETL 1, flip angle 15°, slice thickness 1.5 mm, 12 mm thickness for minIP, matrix 256 × 208 and field of view 240 × 195, and excitation 1 and total acquisition time of 4 minutes and 23 seconds. MRI of brain revealed an irregular thin ring enhancing lesion with restricted diffusion measuring approximately 25 mm × 25 mm. The enhancing portion demonstrated signals similar to the white matter on both noncontrast T1 and T2 sequences. Perilesional vasogenic edema was also noted. SWI images demonstrated prominent hypointensity in the wall consistent with hemorrhage (Figure 1). A stereotactic biopsy was obtained to exclude tumor. Culture of the biopsy material grew* Streptococcus viridans*. He was initiated on intravenous ceftriaxone 2 gm IV every 12 hours and oral metronidazole 500 mg every 6 hours for 10 weeks. He made an uneventful recovery and a follow-up imaging obtained after the completion of antibiotics identified resolution of the lesion with residual hemosiderin scar. No primary focus of infection was identified.

### 2.2. Case 2

**Figure 2:**
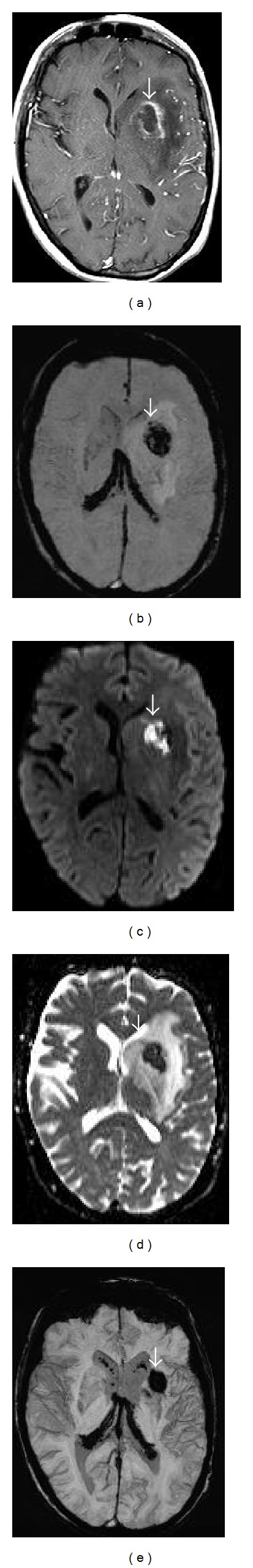
(a) Postgadolinium T1 SE image shows the enhancing wall of the abscess (arrow). Note the prominent surrounding vasogenic edema with mass effect on the left lateral ventricle. (b) Susceptibility weighted image shows profound hypointensity (arrow) in the wall from presence of hemorrhage. Vasogenic edema shows hyperintense signals. (c) Diffusion image demonstrates bright signals due to restricted diffusion from the pus in the center of the abscess (arrow). (d) Apparent diffusion coefficient (ADC) map shows corresponding dark signals confirming true restricted diffusion in the center of the abscess (arrow). (e) Susceptibility weighted image at 6 months followup demonstrates residual hemosiderin in the healed abscess (arrow). Note the resolved edema and mass effect on the left lateral ventricle.

A 59-year-old female with past history of treated myelodysplastic syndrome, basal cell carcinoma, mitral valve prolapse, and melanoma presented to the emergency department with several days history of fatigue, lethargy, slurring of speech, headaches, and altered mental status that started worsening for 3 days prior to admission. On examination, she was febrile. Neurological examination revealed no focal deficits. She had pancytopenia with thrombocytopenia and received blood transfusion every 2 years. Laboratory investigation revealed a total count of 2.32 K/*μ*L, a red blood cell count of 2.39 M/*μ*L, a platelet count of 26 K/*μ*L, prothrombin time 19.7 seconds, INR 1.61, and PTT 39 seconds. Noncontrast CT head obtained at the time of admission identified left basal ganglia hypoattenuating lesion with a central ring like lesion having attenuation similar to the gray matter. No hemorrhage or calcification was seen in the lesion. An MRI of brain was obtained 4 hours after the admission with 1.5T MRI. Along with routine pre- and postcontrast T1 SE, T2 FSE, FLAIR, and diffusion imaging, susceptibility weighted sequence was obtained with parameters TR49 mS, TE40mS, ETL 1, flip angle 15°, slice thickness 1.5 mm, 12 mm thickness for minIP, matrix 256 × 224 with phase encoding steps 155 and field of view 240 × 210, and excitation 1 and total acquisition time of 4 minutes and 23 seconds. MRI of brain revealed an irregular thin ring enhancing lesion in the left anterior lentiform nucleus with associated prominent vasogenic edema. The enhancing portion demonstrated signals similar to the white matter on both noncontrast T1 and T2 sequences. Ring enhancement and restricted diffusion were seen in the lesion with prominent hypointense signals on SWI image in the wall of the lesion (Figure 2). Abscess was considered with a differential of hemorrhagic metastasis. Blood cultures did not grow pathogens. Biopsy was withheld in view of low platelet count. She received intravenous ceftriaxone 2 gm every 12 hours and oral metronidazole 500 mg every 6 hours for a period of 6 weeks. She made an uneventful recovery at the end of antibiotic treatment and follow-up imaging demonstrated resolution of the lesion with residual susceptibility signals from hemosiderin scar.

### 2.3. Case 3

**Figure 3:**
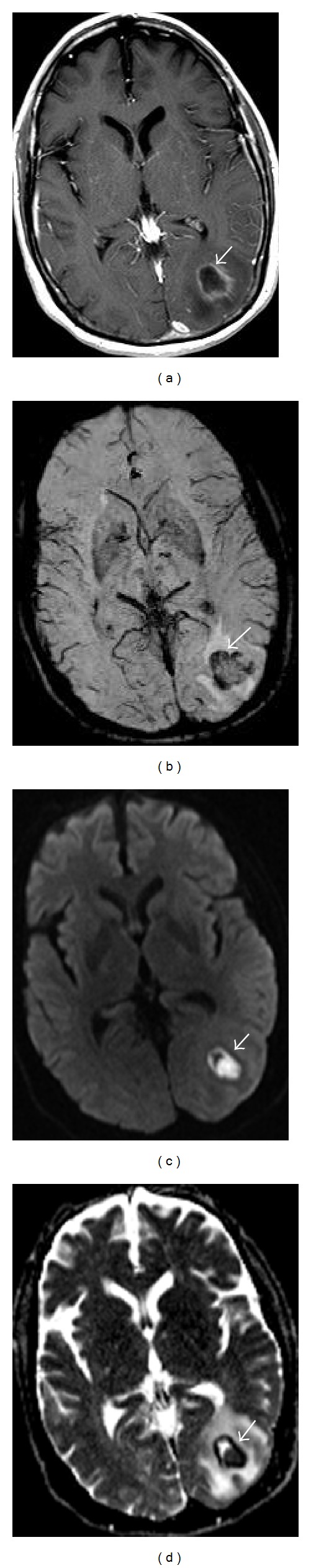
(a) Post gadolinium T1 SE image shows the ring enhancing wall of the abscess (arrow). (b) Susceptibility weighted image shows prominent hypointensity (arrow) in the wall from presence of hemorrhage. (c) Diffusion image demonstrates bright signals due to restricted diffusion from the pus in the center of the abscess. (d) Apparent diffusion coefficient (ADC) map shows corresponding dark signals confirming true restricted diffusion in the center of the abscess.

A 65-year-old male presented to the emergency department with three-day worsening of headache. A neurological assessment revealed no focal deficits. Total count was 9.95 K/*μ*L and platelets were 274 K/*μ*L. His coagulation parameters were not obtained. He did not receive Coumadin. There was no history of bleeding disorder. Noncontrast CT head at the time of admission showed focal hypodense lesion in the left temporooccipital region with no evidence of hemorrhage or calcification. Seven hours after admission, an MRI of brain was obtained as part of evaluation. Along with routine pre- and postcontrast T1 SE, T2 FSE, FLAIR, and diffusion imaging, susceptibility weighted sequence was obtained with parameters TR49 mS, TE40mS, ETL 1, flip angle 15°, slice thickness 1.5 mm, 12 mm thickness for minIP, matrix 256 × 224 with phase encoding steps 155 and field of view 230 × 200, and excitation 1 and total acquisition time of 5 minutes. MRI of brain revealed an irregular thin ring enhancing lesion in the left parietotemporooccipital region with associated perilesional vasogenic edema. The enhancing portion demonstrated signals similar to the white matter on both noncontrast T1 and T2 sequences. Restricted diffusion was seen in the center of the lesion. SWI demonstrated prominent hypointense signals in the enhancing walls (Figure 3). Blood cultures did not grow pathogens. In view of his old age, surgery was done to differentiate tumor from infection. No tumor was identified on histopathological examination. Culture of the surgical tissue grew* Streptococcus viridans*. He received intravenous ceftriaxone 2 gm IV every 12 hours and oral metronidazole 500 mg every 6 hours. Later, his antibiotic was switched to vancomycin, ciprofloxacin, and Flagyl due to drop in blood cell and platelet count. Antibiotics were administered over a period of 6 weeks. Finally, he made an uneventful recovery with resolution of the lesion. No primary source of infection was identified.

## 3. Discussion

In the past, it was considered that a hemorrhagic change in the walls of brain abscess is exceptional [8]. Recently, Gupta et al. used 3T MRI and reported hemorrhage in the wall of brain abscess with 3D T2* susceptibility weighted angiography (SWAN), a variant of susceptibility weighted imaging, in 11 out of 15 cases [6]. In another series, Lai et al. analyzed 14 cases of brain abscess with 1.5T MRI and did not identify hemorrhage in the walls of pyogenic brain abscess on susceptibility weighted imaging [7]. The authors identified only minimal susceptibility signals in the wall of abscesses and thought the susceptibility signals originated from free radicals [7]. However, we identified very prominent hypointense signals on SWI in the enhancing walls of brain abscess in all three of our cases with 1.5T MRI. Central nonenhancing portion demonstrated restricted diffusion from pus in all 3 cases. Although we do not have histological proof to support the presence of hemorrhage, prominent hypointense signals on SWI in our cases unequivocally indicate the presence of hemorrhage. Indeed no calcification was noted and no macroscopic hemorrhage could be recognized in the CT head obtained few hours earlier in all 3 cases. Presence of hemorrhage in our first case made us think of the possibility of metastasis in view of past history of melanoma; however, the presence of restricted diffusion in the ring enhancing lesion led us to consider abscess over hemorrhagic metastasis. The patient received treatment with antibiotics leading to resolution of the lesion after few weeks. Curiously in all of the three of our cases, we noted that the ring enhancement was irregular and thin without the smooth appearance seen in majority of brain abscess. It may be too early to suggest a relationship between identifying hemorrhage in the wall of abscess and irregular enhancement, but it is worth exploring with further studies. Susceptibility signals in the walls of ring enhancing brain lesions may potentially lead to a false positive diagnosis of tumor. It is essential that one should pay adequate attention to the diffusion-weighted sequence to identify restricted diffusion and brain abscess. In pyogenic brain abscess restricted diffusion is attributed to high viscosity of the pus [9]. Majority of ring enhancing brain lesions with restricted diffusion are considered to be brain abscess. In rare instances, metastatic ring enhancing brain lesion has been shown to demonstrate restricted diffusion [10]. MR spectroscopy may hold advantage in these situations to identify amino acids, succinate, and acetate metabolites to provide supportive diagnosis of abscess [11]. It is essential that one should actively search for supportive findings like positive blood culture or presence of septic focus which will point to a diagnosis of brain abscess. If the diagnosis is not settled yet, one may resort to stereotactic biopsy or follow-up imaging after a course of antibiotics.

Susceptibility weighted image is a high resolution 3D fully velocity compensated gradient echo sequence [12]. Its variants include T2* susceptibility weighted angiography (SWAN) and venous blood oxygen level dependent (venoBOLD) techniques. In SWI, data from phase and magnitude images is used to create minimum intensity projection (minIP) image to increase the conspicuity of hemorrhage, clot, and deoxyhemoglobin in veins. This enhances the sensitivity of susceptibility weighted imaging to detect hemorrhage much better than traditional 2D gradient echo T2* sequence [7]. Presence of prominent hypointense signals in the ring enhancing lesions on susceptibility imaging in all three of our cases is consistent with hemorrhage. A histopathological analysis may be the best step to confirm the presence of hemorrhage in the wall of a brain abscess. However, it is important to understand that surgical intervention is not needed in each and every case of brain abscess and smaller lesions can be treated with antibiotics alone.

The pathophysiology of hemorrhage in the wall of brain abscess is not completely understood. Probably it occurs due to fragility of the new capillaries formed in the wall of brain abscess. Increasing mass effect perhaps leads to rupture of the vessel wall and bleeding [2]. Alternatively, lack of thrombosis in the new capillaries could result in rupture of the vessel wall as the abscess expands [3]. Some consider free radical damage to neovascularity from free radicals [4]. Based on experimental and human studies, Britt et al. classified brain abscess into 4 stages including early cerebritis (1–3 days), late cerebritis (4–9 days), early capsule (10–13 days), and late capsule formation (14 days and more) [13]. The authors reported increased neovascularity and capillaries particularly in the late cerebritis stage. Brain abscess may demonstrate petechial hemorrhages in the cerebritis stage [14]. It is possible that they are retained in the capsular stages and become visible on susceptibility weighted imaging. Although our second case had very low platelet count, lack of macroscopic bleeding in brain and similar imaging appearance of the brain abscess to other two cases indicate that a similar mechanism is responsible for susceptibility signals in the wall of this lesion.

Brain abscess represents a significant medical problem, accounting for one in every 10,000 hospital admissions in the United States and remains a serious situation despite recent advances made in detection and therapy [15]. Although the mortality rate has now decreased to 55–20%, rupture of abscess into a ventricle may carry high mortality of up to 80% [16]. Susceptibility sequence is increasingly used in the assessment of brain and it may demonstrate prominent susceptibility signals from hemorrhage in the wall of brain abscess. Prompt identification of brain abscess is essential and appropriate treatment is undertaken to prevent the associated morbidity and mortality.

## 4. Conclusion

Susceptibility weighted sequence at 1.5T MRI can demonstrate prominent hypointense signals from hemorrhage in the walls of pyogenic brain abscess. It is essential that one should pay attention to identifying restricted diffusion and preventing a misdiagnosis of hemorrhagic tumor and delay in the diagnosis of brain abscess. Appropriate clinical background and blood culture should be sought after and, if needed, MR spectroscopy may be utilized to confirm abscess. Prospective analysis with susceptibility weighted imaging at 1.5T MRI will help identify the true incidence of hemorrhage in the wall of pyogenic brain abscess.

## References

[1] Terakawa Y, Takami T, Yamagata T, Saito T, Nakanishi N (2007). Magnetic resonance imaging of brain abscess with hemorrhage: implications for the mechanism of hemorrhage—case report. *Neurologia Medico-Chirurgica*.

[2] Orita T, Fujii M, Hayashi M, Fudaba H, Aoki H (1987). Brain abscess with hemorrhage. *Neuroradiology*.

[3] Devi BI, Bhatia S, Kak VK, Bantwal S, Bhagwati SN (1993). Spontaneous haemorrhage associated with a brain abscess. *Child's Nervous System*.

[4] Kaplan M, Topsakal C, Cihangiroglu M (2005). Hemorrhage into the brain abscess cavity with Fallot's tetralogy. *Pediatric Neurosurgery*.

[5] Sudhakar KV, Agrawal S, Rashid MR, Hussain N, Hussain M, Gupta RK (2001). MRI demonstration of haemorrhage in the wall of a brain abscess: possible implications for diagnosis and management. *Neuroradiology*.

[6] Gupta RK, Tomar V, Awasthi R (2012). T2*-weighted MR angiography substantially increases the detection of hemorrhage in the wall of brain abscess: implications in clinical interpretation. *Neuroradiology*.

[7] Lai P-H, Chang HC, Chuang TC (2012). Susceptibility-weighted imaging in patients with pyogenic brain abscesses at 1.5T: characteristics of the abscess capsule. *The American Journal of Neuroradiology*.

[8] Haimes AB, Zimmerman RD, Morgello S (1989). MR imaging of brain abscesses. *American Journal of Roentgenology*.

[9] Leuthardt EC, Wippold FJ, Oswood MC, Rich KM (2002). Diffusion-weighted MR imaging in the preoperative assessment of brain abscesses. *Surgical Neurology*.

[10] Hartmann M, Jansen O, Heiland S, Sommer C, Münkel K, Sartor K (2001). Restricted diffusion within ring enhancement is not pathognomonic for brain abscess. *American Journal of Neuroradiology*.

[11] Pal D, Bhattacharyya A, Husain M, Prasad KN, Pandey CM, Gupta RK (2010). In vivo proton MR spectroscopy evaluation of pyogenic brain abscesses: a report of 194 cases. *The American Journal of Neuroradiology*.

[12] Barnes SRS, Haacke EM (2009). Susceptibility-weighted imaging: clinical angiographic applications. *Magnetic Resonance Imaging Clinics of North America*.

[13] Britt RH, Enzmann DR, Yeager AS (1981). Neuropathological and computerized tomographic findings in experimental brain abscess. *Journal of Neurosurgery*.

[14] Brown E, Gray F, Love S, Louis DN, Ellison DW (2008). Bacterial infections. *Greenfield’s Neuropathology*.

[15] Townsend GC, Scheld WM (1998). Infections of the central nervous system. *Advances in Internal Medicine*.

[16] Mathisen GE, Patrick Johnson J (1997). Brain abscess. *Clinical Infectious Diseases*.

